# Earliest ontogeny of early Cambrian acrotretoid brachiopods — first evidence for metamorphosis and its implications

**DOI:** 10.1186/s12862-018-1165-6

**Published:** 2018-04-02

**Authors:** Zhiliang Zhang, Leonid E. Popov, Lars E. Holmer, Zhifei Zhang

**Affiliations:** 1 Shaanxi Key laboratory of Early Life and Environments and Department of Geology, State Key Laboratory of Continental Dynamics, Northwest University, Xi’an, 710069 China; 2 Uppsala University, Department of Earth Sciences, Palaeobiology, Villav 16, SE-752 36 Uppsala, Sweden; 3 Department of Geology, National Museum of Wales, Cathays Park, Cardiff, CF10 3NP UK

**Keywords:** Earliest ontogeny, Metamorphosis, Acrotretoidea, Early Cambrian, Heterochrony, South China

## Abstract

**Background:**

Our understanding of the ontogeny of Palaeozoic brachiopods has changed significantly during the last two decades. However, the micromorphic acrotretoids have received relatively little attention, resulting in a poor knowledge of their ontogeny, origin and earliest evolution. The uniquely well preserved early Cambrian fossil records in South China provide a great new opportunity to investigate the phylogenetically important ontogeny of the earliest acrotretoid brachiopods, and give new details of the dramatic changes in anatomy of acrotretoid brachiopods during the transition from planktotrophic larvae to filter feeding sedentary juveniles.

**Results:**

Well preserved specimens of the earliest Cambrian acrotretoid brachiopods *Eohadrotreta zhenbaensis* and *Eohadrotreta*? *zhujiahensis* (Cambrian Series 2, Shuijingtuo Formation, Three Gorges area, South China) provide new insights into early acrotretoid ontogeny, and have significance for elucidating the poorly understood early phylogeny of the linguliform brachiopods. A more comprehensive understanding of the applied terminology based on new observation, especially in definition of the major growth stages (embryo, planktotrophic larva, post-metamorphically sessile juvenile and adult), is established. The so-called acrotretoid “larval shell” of both valves of *Eohadrotreta* demonstrates evidence for metamorphosis (shedding of the larval setae and transitions of shell secretion), during the planktotrophic stage. Therefore, it is here termed the metamorphic shell. The inferred early acrotretoid larval body plan included a bivalved protegulum, secreted at the beginning of the pelagic stage, which later developed two pairs of larval dorsal setal sacs and anterior–posterior alignment of the gut during metamorphosis.

**Conclusion:**

The primary larval body plan of acrotretoid *Eohadrotreta* is now known to have been shared with most early linguliforms and their relatives (including paterinates, siphonotretoids, early linguloids, the problematic mickwitziids, as well as many early rhynchonelliforms). It is suggested that this type of earliest ontogeny can be considered as plesiomorphic for the Brachiopoda and probably first evolved in stem group brachiopods with subsequent heterochronic changes.

## Background

The outstanding early Cambrian fossil Lagerstätten of South China have had a major impact on our current understanding of the first brachiopods and other lophophorates; in particular, new information on their soft body anatomy have been most valuable in tracing the initial radiation of major brachiopod and lophophorate clades [1–6]. However, the micromorphic acrotretoids have received relatively little attention in these studies [7–11]. The group first emerged in the fossil record together with other early lingulates in the Unnamed Cambrian Stage 3, and went extinct in mid Devonian [2, 8]. Consequently, their origin, earliest evolution and ontogeny are still poorly known, and can be examined only based on the fossil record.

The larval developments of living linguloid [12, 13], discinoid [14, 15], terebratulid [16–19] and craniiform [20] brachiopods have been well documented with detailed accounts of their embryogenesis, fertilization, free swimming stage, and larval settlement.

Numerous previous studies have also shown that it is possible to discriminate planktotrophic and lecithotrophic larval life histories in the earliest ontogenies of fossil brachiopods, including extinct linguliforms [7, 21–30], craniiforms [31, 32] and rhynchonelliforms [32–38].

The main objective of this paper is to provide the first detailed account of the earliest ontogenetic development for two acrotretoid species *Eohadrotreta zhenbaensis* and *Eohadrotreta*? *zhujiahensis* from the lower Cambrian Shuijingtuo Formation of the Three Gorges area in South China, which are among the earliest known representatives of the Acrotretoidea. Our results indicate that the acrotretoids had a metamorphic shell with two pairs of dorsal setal sacs and a straight anterior–posterior gut alignment – a type of ontogeny that has already been recorded from other early Cambrian brachiopods and carrying important implications for the understanding of their phylogeny and relationship to other brachiopod lineages. Detailed information on the post-metamorphic shell growth patterns of these species have been recently investigated [39].

## Methods

**Fig. 1:**
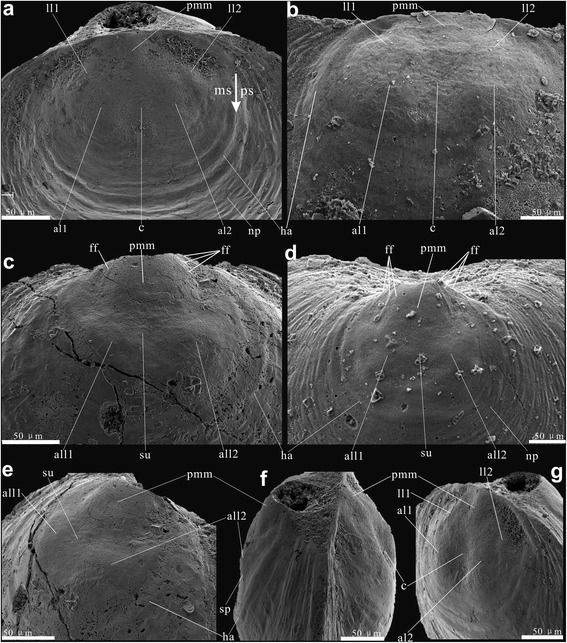
Earliest ontogenetic characters on both dorsal and ventral valves of *Eohadrotreta zhenbaensis* from South China. **a**, **f**, **g** conjunct juvenile valves, showing the protegulum and two pairs of lobes on metamorphic shell; (**a**) dorsal view, noting the metamorphic shell and post-metamorphic shell bounded by the halo (arrow); (**f**) lateral view; (**g**) lateral dorsal view. **b** protegulum and two pairs of lobes on metamorphic shell of a larger dorsal valve. **c**, **e** conjunct juvenile valves; (**c**) ventral view, noting the protegulum and a pair of lobes on metamorphic shell; (**e**) enlarged ventral metamorphic shell noting the median sulcus. **d** protegulum, median sulcus and paired lobes on metamorphic shell of a larger ventral valve. Abbreviations: al, anterior lobe; all, anterolateral lobe; c, shallow cleft; f, pedicle foramen; ff, fine fold; ha, halo; ll, lateral lobe; ms, metamorphic shell; np, nick points; pmm, posteromedian mound; ps, post-metamorphic shell; sp., slope; su, median sulcus. Scale bars: 50 μm

All specimens described and illustrated here are from the Shuijingtuo Formation at Aijiahe section, Sandouping County in the Three Gorges area, South China ([40], Figure 1). The geological and geographical setting was described in detail by [5, 40]. A total of 75 conjunct valves, 2536 ventral valves and 2517 dorsal valves of *E. zhenbaensis*, and 23 conjunct valves, 102 ventral valves and 115 dorsal valves of *E.*? *zhujiahensis* were found in the middle and upper part of the Shuijingtuo Formation. The specimens were dissolved from limestones by applying 7% concentrated acetic acid, 63% water and 30% buffering solution formed after the dissolution of previous samples to avoid chemical damage, as proposed by [41]. Scanning electron microscope (SEM) images of uncoated fossils were taken with a Philips Fei Quanta 400-FEG with 20 KV and 70–80 Pa at State Key Laboratory of Continental Dynamics, Northwest University, Xi’an. Some uncoated specimens were taken to Sweden and SEM imaging was carried out with a Zeiss Supra 35 VP field emission with 5 KV at the Evolutionary Biology Centre of Uppsala University. No permissions were required for collecting of the fossils discussed in the study from the Shuijingtuo Formation at the Aijiahe section, Sandouping County in the Three Gorges area of South China, which complied with all relevant regulations in China.

### Terminology

The terminology presently used for describing the early ontogenetic stages of brachiopods has varied significantly from one publication to other, and in particular common terms – including, the protegulum, embryonic shell, first formed shell, larval shell, etc. – have been used in widely different ways (compare, e.g. [24–27, 32, 38, 42]).

Moreover, our understanding of the ontogeny of Palaeozoic brachiopods has changed significantly in the last two decades, since the first brachiopod volume of the revised ‘Treatise on Invertebrate Paleontology, Part H, Brachiopoda’ [43] was published. The simplistic concept that recent (and probably extinct) linguliforms are all planktotrophic, while craniiforms and rhynchonelliforms are lecithotrophic was challenged first by [27, 35], who advocated that lecithotrophy had evolved only in the crown group rhynchonelliform brachiopods. Subsequently planktotrophic larvae were described in the orthoids [33], strophomenoids [44], orthotetidines [38, 44], polytoechioids [36]; however, at least some orthoids [34], clitambonitoids [36] and triplesioids were most likely characterised by lecithotrophic larvae, which probably evolved independently from those in the crown group rhynchonelliforms.

Lüter [45] pointed to the fact that lingulids are characterised by having a direct development, and thus lost a ‘true’ larval stage. Indeed, the planktotrophic larvae of Early Palaeozoic brachiopods, including both linguliforms and rhynchonelliforms, have little in common with living juvenile lingulids at their free swimming stage (e.g. [24–26, 28, 29, 31, 32, 46–48]). Thus, a more comprehensive discussion of the applied terminology, especially in definition of the major growth stages (embryo, planktotrophic larva, post-metamorphically sessile juvenile and adult) is needed. The most recent reviews of the terminology currently applied to the morphological characters observed on juvenile brachiopod shells can be found in several publications e.g. [31, 35, 38, 39, 42], but here we follow the terminology of the two papers [38, 39].

The earliest shell, identifiable through the brachiopod ontogeny, is called protegulum. The term was first introduced by [49] and traditionally was defined (e.g. [43]) as the ‘first-formed shell of periostracum and mineralised lining secreted simultaneously by both mantles’. However, this definition is problematic due to the fact that there are a large number of brachiopods (e.g. craniids, strophomenates, siphonotretoids), in which the secretion of the ventral valve is considerably delayed towards the end of metamorphosis. Therefore, only the dorsal protegulum can be observed [28, 31, 32, 36, 38, 46]. Also the mineralized lining of the protegulum may be a characteristic only of more derived brachiopods, where the lecithotrophic larvae form a mineralized shell at the early stage of metamorphosis, e.g. crown group rhynchonelliforms [17, 18], clitambonitoids [36] and triplesioids [50]. By contrast, the juvenile shells of the Early Palaeozoic brachiopods with planktotrophic larvae exhibit distinct traces of plastic deformations (e.g. [26, 28, 36, 48]), suggesting that the shell, formed soon after hatching from the vitelline membrane, was only loosely mineralized, or more likely, entirely organic. Moreover, in living *Lingula*, the shell is first secreted as an organic membrane at the end of the embryonic stage before hatching [12]. With this in mind, Chuang [15] proposed to restrict protegulum only to the lingulids. However, unlike any other brachiopods, living lingulids are characterised by having a direct development [45], which should be considered as a relatively recent novelty. To date, the earliest known lingulids with embryonic shell (in the sense of [12]) are documented from the Cretaceous ([24], Figure 4; [51], Figure 4F-H) and probably also from the Late Jurassic [52]. The so-called dorsal and ventral ‘embryonic’ shells of the Devonian lingulide *Lingulipora* [25], show the presence of nick points, which could only have been formed at the mantle margin and after metamorphosis. Freeman and Lundelius [27, 35] indicated the presence of embryonic shells in a number of distantly related extinct brachiopod taxa. However, the only applied criterion for identifying embryonic shells was that the shell size at formation of the halo should not exceed the maximum diameter of the brachiopod egg. This simple criterion is problematic, since in recent *Lingula*, the maximum width of the embryonic shell approaches 150 μm [12], while the size of the protegulum in lecithotrophic larva of *Novocrania* is only 60 μm [20].

In Early Palaeozoic brachiopods with planktotrophic larvae, the protegulum often can be seen as a mound surrounded by elevated paired lobes. This type of larva was first described from the paterinates ([26], text-Figure 3); however, the protegulum was mistakenly referred to as the alimentary mound, but was later reconsidered as a rudiment of the protegulum [28, 36, 47, 48].

The protegulum is here defined as the first formed shell, which is secreted post-embryonically, simultaneously by the outer epithelium of the larval mantle lobes of a planktotrophic larva [15, 26, 28, 32, 38, 42, 53], or by the whole surface of dorsal or dorsal and ventral mantles of a lecithotrophic larva [17, 18, 20, 31, 32] during the early stage of metamorphosis.

Data on the ontogeny of recent craniids [20] and rhynchonelliforms [17, 18] convincingly demonstrated that the protegulum formed during early metamorphosis, within 24 h after settlement. The continuous growth of the shell occurs without interruption until the end of metamorphosis. Therefore, the protegulum on the surface of the mature shells is often difficult to recognize, and the first distinct halo is formed on completion of metamorphosis, when the conveyor-belt system sensu [42] of the shell secretion starts to work. Thus the halo, which is a distinct growth mark indicating a substantial change in the shell secretion, often shows not the outer boundary of the protegulum, but outlines the shell on completion of metamorphosis. In *Novocrania*, the size of a fully grown metamorphic shell, defined by the halo, approaches 300 μm. It significantly exceeds the size of the protegulum observed by [20].

The brephic shell, growing incrementally during the late stage of metamorphosis, encloses the protegulum [34, 35, 42, 48, 53, 54]. Its outer boundary is marked by the following characters: (1) fundamental changes in shell secretion, with initiation of the conveyor-belt system and related differentiation of the shell fabric into primary and secondary layers are established by that time; (2) formation of nick points and characters of radial ornament indicating the presence of marginal mantle setae and related musculature; (3) shedding of the larval setae (sensu [45]). The metamorphic shell, formed by completion of metamorphosis, here includes the simultaneously secreted protegulum, the incrementally growing brephic shell, and it is bounded by the halo (Fig. 1).

As first pointed out by [26], the presence of larval (or embryonic) setae in planktotrophic larvae of the early Palaeozoic brachiopods can be indicated by 1–3 pairs of symmetrical lobes on the dorsal valve located within the halo [28–30, 46–48]. These lobes represent the impressions of setal sacs, which originated when the growing organic or weakly mineralised brephic shell overlapped the larval setal sacs during the free swimming stage of planktotrophic larval phase. Following [45], we consider the terms ‘larval setae’ and ‘larval setal sacs’ as preferable in application to these features.

The metamorphic shell, as here defined, is identical to the first formed shell as used by [36, 39, 54], but not as it was used in [42], and also not to the protegulum, as defined by [43]. The shell outside the halo, which exhibits concentric growth marks disrupted by nick points and other ‘adult’ surface features, is referred here as post-metamorphic or adult shell. We restrict the term neanic shell, to the growth stage after metamorphosis was completed, as suggested by [28, 43], and this stage involves the formation of post-metamorphic shell, including a regime of adult shell secretion and development of mantle marginal setae. The neanic shell is characterised by growth disturbances, produced by the growing mantle lobes, it may not exhibit all adult surface features (e.g. nick points and drapes). The mature shell, surrounding the neanic shell, is characterized by the development of all adult surface features including those that normally distinguish genera and species [42, 53]. The post-metamorphic shell, formed after metamorphosis, includes, as here defined, the neanic shell and the mature shell (Fig. 1a). In that sense, the shell formed during the pelagic stage in recent lingulids and discinids should be considered as the neanic shell and therefore it cannot be compared directly with the metamorphic shell grown by the planktotrophic larvae of the Early Palaeozoic linguliforms and rhynchonelliforms.

The terminology here applied to the *Eohadrotreta* post-metamorphic shell morphology is the same as applied by [39, 40, 55]. Abbreviations used in the text and on the figures are: al, anterior lobe; all, anterolateral lobe; bs, brephic shell; c, shallow cleft; f, pedicle foramen; ff, fine fold; ha, halo; ll, lateral lobe; lse, larval setae; mas, mature shell; ms, metamorphic shell; mse, marginal mantle setae; ns, neanic shell; np, nick points; pr, protegulum; pmm, posteromedian mound; ps, post-metamorphic shell; sp., slope; su, median sulcus; te, tentacle.

## Results

### Outline of acrotretoid juvenile shell morphology

**Fig. 2:**
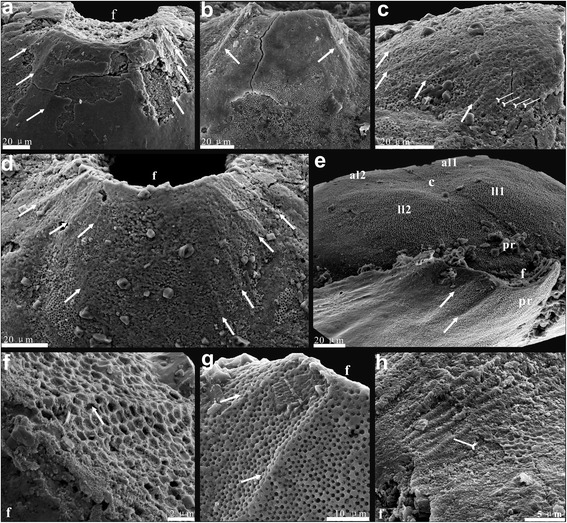
Fine folds on ventral protegulum and ridges on ventral brephic shell of *Eohadrotreta*? *zhujiahensis* and *Eohadrotreta zhenbaensis*. **a** enlarged protegulum of *E.*? *zhujiahensis*, showing three pairs of fine folds by arrows. **b**-**h** *E. zhenbaensis*; (**b)** a pair of thin folds (arrows) on a lager specimen; (**c**-**d**) fine folds on ventral protegulum; (**c**) lateral view, showing thin folds by arrows and densely packed ridges by tailed arrows; (**d)**, show four pairs of folds by arrows; (**e**) conjunct valves, show the fine folds of ventral protegulum by arrows; (**f**) enlarged view, noting the thin fold covered by the above pitting structures; (**g**) enlarged view of (**e**); (**h**) enlargement of the ridges (tailed arrows) covered by the pitting structures on the posterior margin of brephic shell. Abbreviations: al, anterior lobe; c, shallow cleft; f, pedicle foramen; ll, lateral lobe; pr, protegulum. Scale bars: a-e, 20 μm; f, 2 μm; g, 10 μm; h, 5 μm

**Fig. 3:**
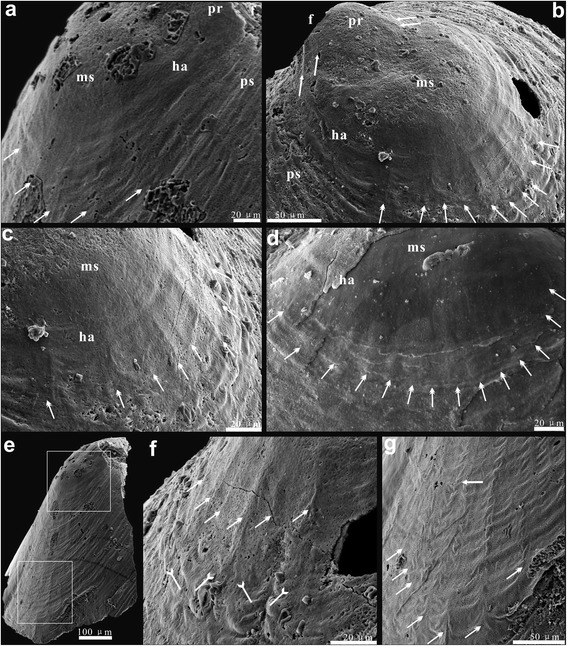
Growth disturbances caused by marginal mantle setae, on both ventral and dorsal post-metamorphic shells of *Eohadrotreta zhenbaensis*. **a**, five bunches of pronounced ridges and associated narrow median troughs on ventral mature shell (double-headed arrows). **b**-**c** ventral valve; (**b**) general view of a juvenile specimen showing two pairs of fine folds on protegulum by arrows and ten bunches of ridges with narrow median troughs by double-headed arrows; (**c**) detailed view, show ridges extending anteriorly from the margin of the halo. **d** fourteen bunches of ridges with associated nick points on dorsal mature shell by double-headed arrows**. e** lateral view showing the pronounced drapes interrupted by nick points, and ridges on ventral post-metamorphic shell, the upper box indicates the area shown in (**a**) and lower box indicates area shown in (**g**). **f** demonstrate ridges by double-headed arrows and the related nick points by tailed arrows. **g** the continuous distribution of the nick points extending to the margin of post-metamorphic shell by double-headed arrows, noting the divergence of ridges by arrow. Abbreviations: ha, halo; ms, metamorphic shell; pr, protegulum; ps, post-metamorphic shell. Scale bars: a, c-d, f, 20 μm; b, g, 50 μm; e, 100 μm

**Fig. 4:**
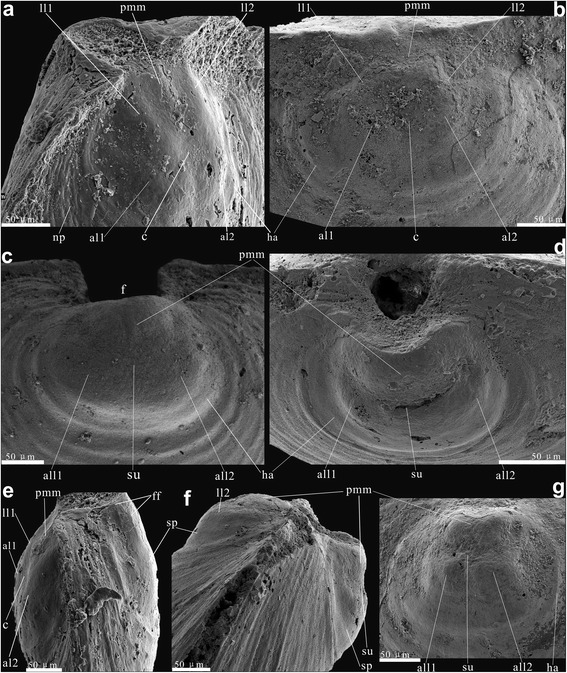
Earliest ontogeny characters on both ventral and dorsal valves of *Eohadrotreta*? *zhujiahensis* from South China. **a**, **e** conjunct valves; (**a**) lateral dorsal view, showing the protegulum and two pairs of lobes on metamorphic shell; (**e**) lateral view. **b**, **g** protegulum and two pairs of lobes on metamorphic shell of a larger conjunct valves; (**b**) dorsal view; (**g**) ventral view. **c** protegulum and a pair of lobes on metamorphic shell of ventral valve. **d**, **f** conjunct valves; (**d**) ventral view, showing protegulum, median sulcus and paired lobes on metamorphic shell; (**f**) lateral view, noting the pronounced slop on the anterior margin of metamorphic shell. Abbreviations: al, anterior lobe; all, anterolateral lobe; c, shallow cleft; f, pedicle foramen; ff, fine fold; ha, halo; ll, lateral lobe; np, nick points; pmm, posteromedian mound; sp., slope; su, median sulcus. Scale bars: 50 μm

Fine details of the dorsal and ventral valve morphology of the umbonal area are best preserved in juvenile specimens of *Eohadrotreta zhenbaensis* and *Eohadrotreta*? *zhujiahensis*. Many early ontogenetic features can also be observed on larger specimens. General patterns of early shell growth are very similar in both species, while there are also some minor, but consistent differences discussed in greater details below. The pitted area in both valves of the acrotretoid shell, often described as ‘larval shell’, is referred to here as the metamorphic shell (Figs. 1, 2, 3 and 4). It is bounded by the distinct halo, suggesting secretion of the post-metamorphic shell started outside the halo. The metamorphic shell was essentially organic in composition when first secreted, and had enough flexibility to cast some important anatomical features of the larval body before it was sufficiently mineralised. Importantly, in both valves the metamorphic shell are preserved as casts on the outer surface of the secondary layer of calcium phosphate secreted at the initial stage of post-metamorphic growth.

### Juvenile shell growth in *Eohadrotreta*

As in other acrotretoids, there is a distinct subcircular area on average 220 μm wide at the dorsal umbo (Table 1). It bears micro-ornament of densely spaced hemispherical pits and is surrounded by the halo, which is considered here as the outer boundary of the metamorphic shell. The posteromedial part of the area, about 40–65 μm long (about 30% metamorphic shell length on average; Table 1) and 60–78 μm wide, is gently inflated and surrounded by a shallow and narrow, subcircular depression (Fig. 1a–b). This posteromedial mound is considered here as a rudiment of the protegulum by analogy to a similar structure observed on the dorsal metamorphic shell of many other early Palaeozoic brachiopods (e.g. [28, 36, 46–48]). Laterally and anteriorly to the protegulum, there are two pairs of symmetrically placed inflated lobes (Fig. 1a, f–g) with the anterior pair divided by a shallow cleft. Similar structures on the metamorphic shell of other early Palaeozoic brachiopods have been repeatedly described as impressions of the larval setal sacs (e.g. [26, 28, 29, 36, 46–48]) and this interpretation is also accepted here. The outer margins of the metamorphic shell in front of the setal sac impressions are gently inclined peripherally and covered by discontinuous concentric wrinkles superimposed on the pitted micro-ornament, which represent the clear evidence of a peripheral growth of the metamorphic shell (Fig. 1a). The pitted ornament is gradually fading towards the halo.

**Table 1:** Average dimensions and ratios of ventral and dorsal valves of *Eohadrotreta zhenbaensis* from the Cambrian (Series 2) Shuijingtuo Formation of the Three Gorges area, South China

Ventral valve	L_pr_	W_pr_	L_ms_	W_ms_	L_f_	W_f_	L_pr_/W_pr_	L_ms_/W_ms_	L_pr_/L_ms_	W_pr_/W_ms_
N	23	23	61	67	23	24	23	59	21	23
Mean	47	71	173	206	53	65	66.94%	84.76%	25.95%	32.27%
Min	40	58	125	146	25	52	53.25%	68.15%	20.48%	25.88%
Max	55	79	213	282	77	83	77.59%	97.52%	34.87%	42.53%
Median	48	70	173	203	52	65	66.23%	85.71%	25.81%	32.46%
SD	4	5	24	29	14	8	7.13%	6.80%	3.52%	3.61%
Dorsal valve	L_pr_	W_pr_	L_ms_	W_ms_	L_pr_/W_pr_			L_ms_/W_ms_	L_pr_/L_ms_	W_pr_/W_ms_
N	19	7	32	34	6			31	17	6
Mean	53	71	166	220	74.25%			74.96%	29.57%	33.77%
Min	40	60	114	186	60.00%			55.38%	21.14%	27.76%
Max	65	78	246	274	82.35%			93.90%	38.56%	41.94%
Median	53	74	159	214	74.55%			77.25%	28.82%	33.17%
SD	7	6	32	25	8.15%			10.89%	4.28%	5.24%

All measurements are in μm. Abbreviations: *L*_*pr*_*, W*_*pr*_ length and width of protegulum, *L*_*ms*_*, W*_*ms*_ length and width of metamorphic shell, *L*_*f*_*, W*_*f*_–length and width of pedicle foramen

The metamorphic shell is also easily recognisable on the ventral valve of *E. zhenbaensis*. It is bordered by a distinct halo and ornamented by fine, hemispherical pits. An inflated posteromedial mound and a pair of anterior lobes divided by a median sulcus are easily recognisable within the ventral umbonal area outlined by the halo (Fig, 1c–e). The ventral posteromedial mound is about 40–55 μm long and 58–79 μm wide (average protegulum length/metamorphic shell length 0.26; Table 1). It is comparable to the size of the dorsal protegulum and it is here considered by analogy as a rudiment of the protegulum secreted simultaneously by outer epithelia of the larval mantle lobes during planktotrophic larva stage. The posterior margin of the protegulum is broadly arched and gently concave inwards. Up to four pairs of symmetrically placed fine folds radiating from the posterior shell margin in the area of the pedicle opening are present on the posterolateral flanks of the protegulum (Fig. 2c–d). Their number is varying from specimen to specimen, probably due to varying degrees of exfoliation and distortion (Figs. 1c–d and 2b–e). In well preserved individuals, these folds are covered by regular hemispherical pits; although no distortion or other deformation of the pitted ornament can be seen (Fig. 2f–g). Thus there is no evidence of plastic deformation caused by the moving setae (e.g. nick points) nor are setal follicles present, so their attribution to larval setae looks unlikely. Most probably these fine folds reflect a folded surface of the outer epithelium at the time of secretion of the protegulum. However, their function remains uncertain. Sometimes there are also 4–7 finer densely packed ridges about 5–15 μm long preserved close to the posterolateral margin of the ventral medial mould (Fig. 2c, h). As can be seen in some specimens, these ridges are actually impressed on the outmost lamina of the secondary columnar shell, below the undisturbed outer primary layer bearing pitted ornament (Fig. 2h). A possible explanation is that these ridges were formed at the initial stage of mineralised shell secretion, probably near the end of metamorphosis. Their position may suggest that these disturbances could be produced by spasmodic contraction of the dermal muscles at the pedicle base. Such contraction can deform the plastic zone of the shell along the newly secreted shell margin, probably at the time of settlement. The final transition from the metamorphic to post-metamorphic shell can be seen within strongly peripherally inclined concentric belt surrounding the ventral anterolateral lobes and delineated outwards by the halo. Within this area, the pitted micro-ornament gradually fades towards the outer boundary of the metamorphic shell, while indistinct growth marks gradually emerge (Fig. 1d).

The regular adult growth marks in the shape of drapes interrupted by nick points, ridges and associated median troughs c. 80–100 μm long (Fig. 3a–c, e) are rather common in acrotretoids and attributed, according to [55], to the stress set up by marginal mantle setae in the plastic zone of shell along the mature mantle edge (Fig. 3e–g). Similarly to the ventral valve, the transitional zone from the dorsal metamorphic to post-metamorphic shell is also accentuated by the appearance of the nick points and associated median troughs c. 100 μm long (Fig. 3d) immediately outside the halo. These growth disturbances indicate the presence of marginal mantle setae, while the conveyor-belt shell secretion was commenced already at that stage.

The juvenile shell growth of *Eohadrotreta*? *zhujiahensis* is closely comparable with that of *Eohadrotreta zhenbaensis*. The dorsal metamorphic shell in *E.*? *zhujiahensis* is covered by micro-ornament of regular hemispherical pits. It is also lobate and bounded by a distinct halo (Fig. 4a**–**b, e, g). A protegulum about 37–69 μm long and 62–83 μm wide (average protegulum length/metamorphic shell length 0.31; *N* = 5) occupies the posteromedial position and has a shape of gently inflated mound. Setal sac impressions are represented by two pairs of lobes with the anterior pair more pronounced than in *E. zhenbaensis* (Fig. 4f) and also divided by a median cleft. The ventral metamorphic shell of *E.*? *zhujiahensis* also shows the ventral protegulum in a shape of a posteromedial lobe (Fig. 4c**–**d) and a pair of anterolateral lobes divided by a median sulcus. The periphery of the metamorphic shell in both valves exhibits a narrow transitional zone with gradually fading pits and a few indistinct growth marks, before the secretion of the adult shell commenced (Fig. 4b**–**c).

## Discussion

### Inferred earliest ontogeny of *Eohadrotreta*

**Fig. 5:**
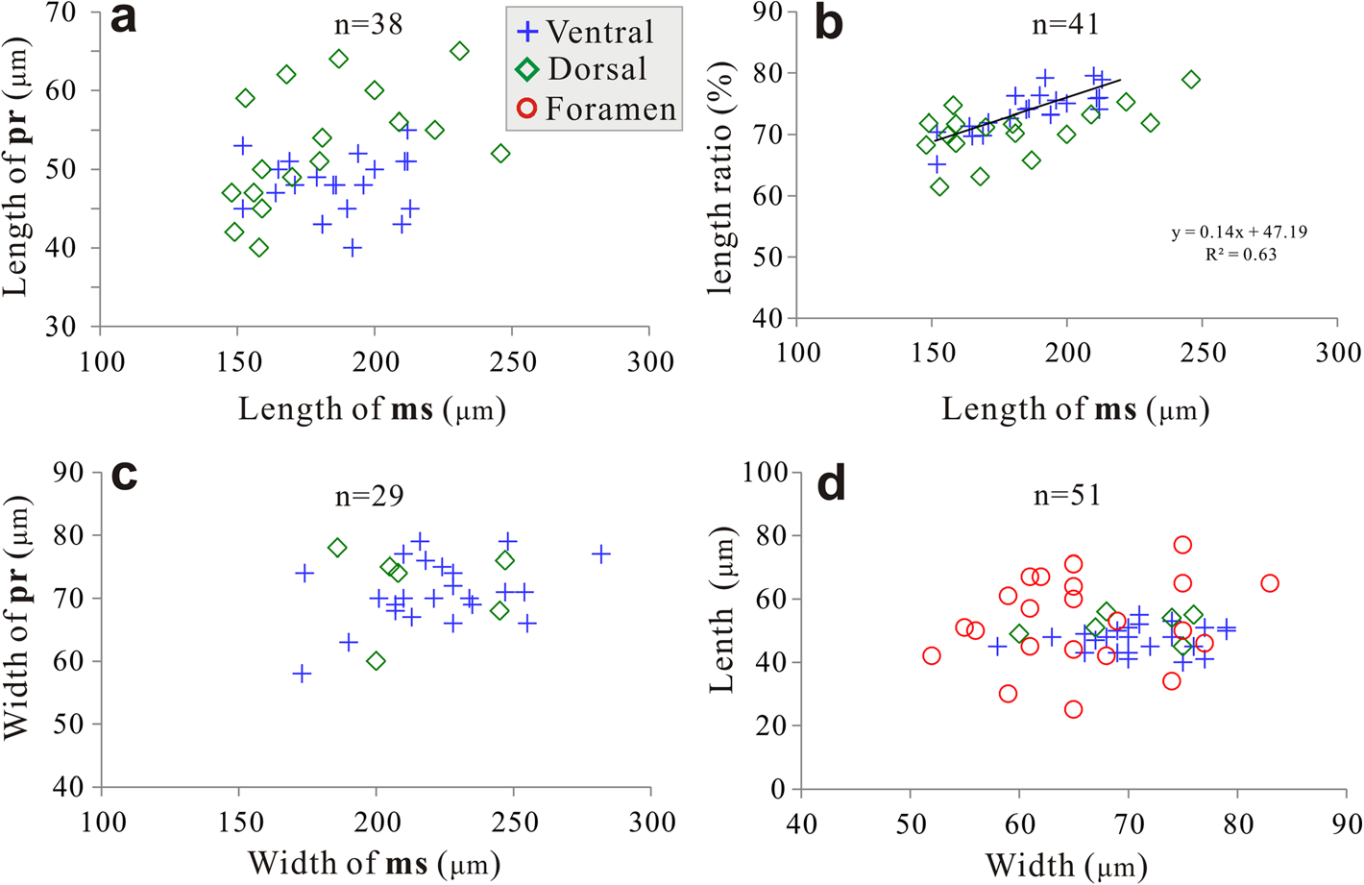
Bivariate plots of ventral and dorsal valves of *Eohadrotreta zhenbaensis* from South China. **a** plots of protegulum length – metamorphic shell length ratio of ventral and dorsal valves. **b** plots of the length ratio of bs/ms (brephic shell / metamorphic shell) – metamorphic shell length ratio of ventral and dorsal valves. **c** plots of protegulum width –metamorphic shell width ratio of ventral and dorsal valves. **d** plots of length– width ratio of protegula of ventral and dorsal valves, and plots of length– width ratio of ventral pedicle foramen. Abbreviations: ms, metamorphic shell; pr, protegulum

The protegulum was secreted initially as a bivalved shell, consisting of two organic membranes, not exceeding 80 μm in width. This can be inferred convincingly from the umbonal shells of both species of *Eohadrotreta* (Figs. 1f and 4f). It is interesting to note that the size of protegula in both ventral and dorsal valves has no direct correlation with the size of the corresponding metamorphic shell (Fig. 5a, c). However, the size of the ventral incipient pedicle opening is comparable to the size of ventral and dorsal protegula, indicating that the rudiment pedicle opening was probably formed during the free swimming stage (Fig. 5d). The broadly arched posterior margin of the ventral protegulum suggests that the dorsal and ventral valves did not come together along the posterior shell margin, and that the larva had a sizable posterior lobe that was not completely covered by shell (Figs. 1a and 4a, e). It is also likely that the lophophore, if it existed at this stage, was not covered by the shell. The subsequent peripheral growth of the brephic shell occurred during the planktotrophic stage, when its size increased to almost 200 μm suggesting the existence of distinct dorsal and ventral mantle lobes. The brephic shell length/ metamorphic shell length ratios of both valves show linear correlation during shell growth, indicating continuous growth of the brephic shell through feeding (Fig. 5b). By that time, the metamorphic shell covered setal sacs on the dorsal side of the larval body and also a lophophore.

**Fig. 6:**
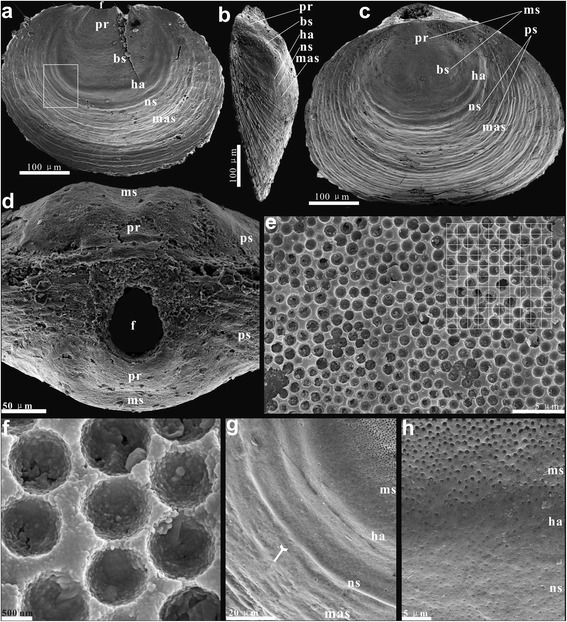
Earliest ontogeny of *Eohadrotreta* from the Cambrian (Series 2) Shuijingtuo Formation, South China. **a** ventral view of conjunct juvenile valves, marking the shell growth stages, box indicates the area shown in (**g)**. **b** lateral view of juvenile dorsal valve, marking the shell growth stages. **c** dorsal view of conjunct juvenile valves, marking the shell growth stages. **d** posterior view of conjunct valves. **e** pitting structures on ventral brephic shell. **f** enlarged pits of (**e**) show uniform size and orderly distributed pattern. **g** gradual transition at the boundary between metamorphic shell and mature shell, marking the nick points of mature shell by tailed arrow. **h** the diminished pitting structures still occur on the halo surface and extend to neanic shell before they eventually vanish. Abbreviations: bs, brephic shell; f, pedicle foramen; ha, halo; mas, mature shell; ms, metamorphic shell; ns, neanic shell; pr, protegulum; ps, post-metamorphic shell. Scale bars: a-c, 100 μm; d, 50 μm; e, h, 5 μm; f, 20 μm; g, 500 nm

The larval settlement probably occurred at a growth stage, when the shell size of the larva exceeded 200 μm; in terms of shell growth it probably corresponded to the inner boundary of the transitional zone inside the halo of the shell, manifesting the beginning of metamorphosis (Fig. 6a–d). Initial settlement occurred on the posterior part of the larval body. By that stage the larval setae, associated with the dorsal setal sacs, were probably lost. The metamorphosis also included axial modifications in the larval body, expressed in the ‘enrolment’ of the ventral valve as discussed by ([56], p. 409, Figure 3). This process probably resulted in changes in the arrangement of muscles controlling the shell movements and the position of the intestine, while the anus moved into an anterolateral position. The post-metamorphic shell outside the halo exhibited all major features of the adult animal including nick points suggesting a development of marginal mantle setae, and secretion of a composite, organophosphatic shell composed of an organic periostracum, mineralised primary and columnar secondary layers ([40], Figure 2). The accretionary shell growth, controlled by the conveyor-belt system, was established by the end of metamorphosis, which is evident from the shell structure and regular growth marks [55].

However, some concentrically cambered stripes usually occur on the neanic shell after the formation of the halo (Fig. 6a, c), until the typical draped structures and nick points of the acrotretoid mature shell start to form (Fig. 6g). Furthermore, the diminished pitting structures also occur on the halo surface and extend on the neanic shell before they are eventually lost (Fig. 6h). Therefore, the significant transitions marked by the halo are most probably linked to metamorphosis including further differentiation of both inner and outer mantle, but are not precisely corresponded to the change of the larval life mode [38, 45, 53]. This is also indicated by the existence of the halo ring found in one pelagic specimen but not in a newly settled juvenile [53].

The umbonal surface of both vales of *Eohadrotreta* is covered by hemispherical pittings, which represent the negative cast of the inner surface of the metamorphic shell (Fig. 6a, e). These pits are about 1.1 μm in diameter with variation from 0.8 to 1.5 μm, having uniform discoidal shape and even distribution (Fig. 6e–f). There are 64 pits in an area of 10 μm × 10 μm (Fig. 6e). So there are about 2500 pits on each surface of both ventral and dorsal metamorphic shells. According to [42], the hemispherical pits covering the protegulum and the entire metamorphic shell were probably built of membrane bound spheroids. Williams argued in favour of probable calcareous mineralisation of these spheroids [42]. Indeed, the observed structure suggests the presence of hard granules enclosed in a proteinaceous matrix. Nevertheless, the existing evidence on their original chemical composition is inconclusive, and there is no good evidence that any kind of shell mineralisation was commenced before the conveyor-belt shell secretion system came into work. Therefore composition of the protegulum and metamorphic shell is considered here as unknown, but probably entirely organic. It is also possible that the composition of spherules was siliceous, by analogy to the siliceous tablets of the Devonian *Schizobolus* ([53], Figure 3 N), Silurian *Opatrilkiella* ([42], plate 2, Figures 11, 12) and extant discinids [53, 57, 58]. Williams did not support this notion [42], but since it is likely that the function of the spherules may have been protection against ultraviolet radiation penetrating surface waters as suggested by [58], this is still a possibility.

### Comparison of brachiopod larval development

**Fig. 7:**
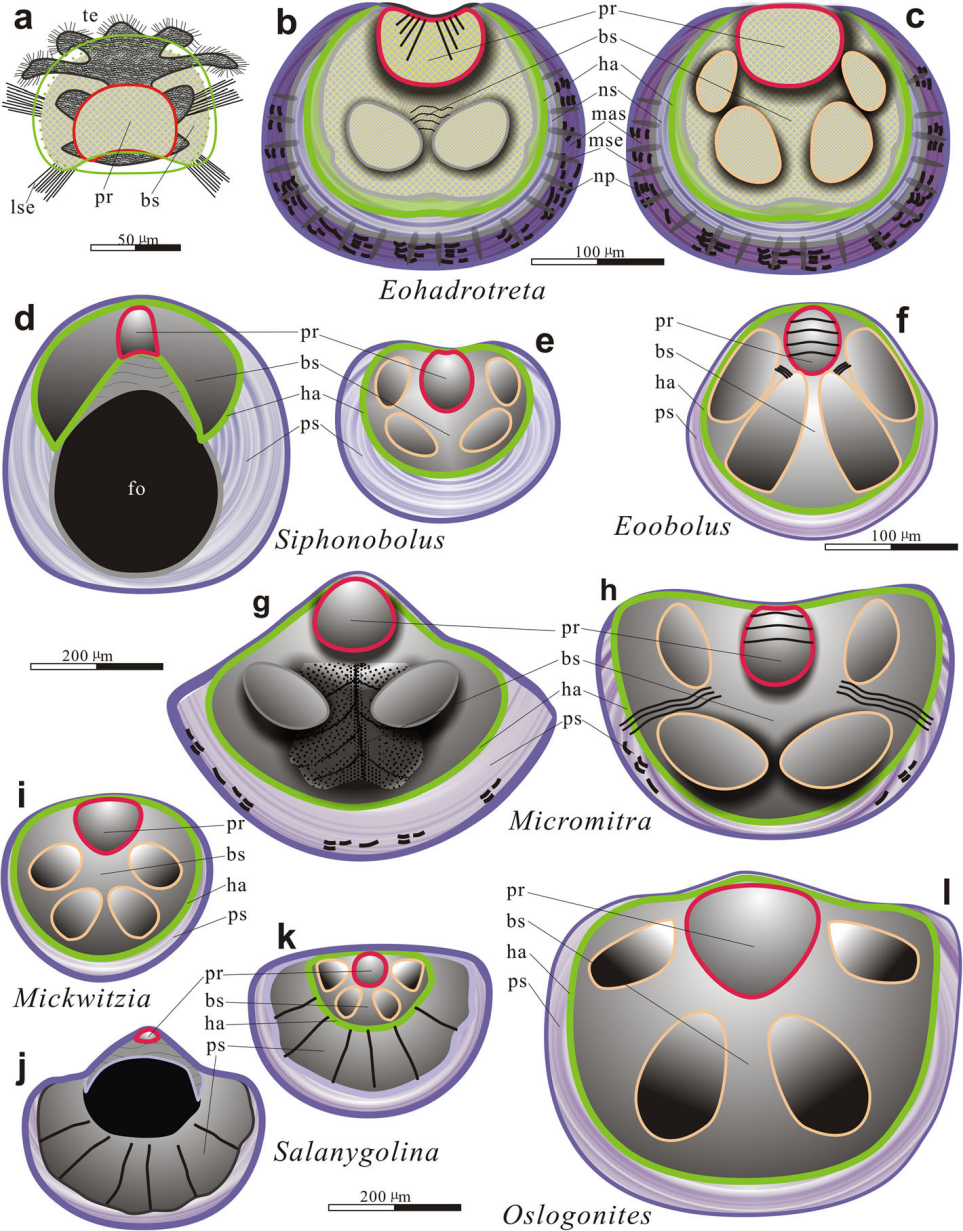
Schematic drawings showing morphology, relative sizes and boundaries of the protegulum, brephic shell, neanic shell and mature shell. **a** planktotrophic larva of *Eohadrotreta* with pitted protegulum, brephic shell and larva setae (modified from [11, 15]). **b**-**c** ventral and dorsal juveniles of *Eohadrotreta* from the Cambrian (Series 2) Shuijingtuo Formation of South China. **d**-**e** ventral and dorsal juveniles of *Siphonobolus* from the Cambrian (Furongian) of Iran (modified from [28]). **f** dorsal juvenile of *Eoobolus* from the Cambrian (Series 2) of Canada (modified from [29]). **g**-**h** ventral and dorsal juveniles of *Micromitra* from the middle Cambrian of Turkey (modified from [26]). **i** juvenile of *Mickwitzia* from Cambrian (Series 2) of Canada (modified from [62]). **j**-**k** ventral and dorsal juveniles of *Salanygolina* from the Cambrian (Series 2) of Mongolia (modified from [46]). **l** dorsal juvenile of *Oslogonites* from Meddle Ordovician of Russian (modified from [36]). Abbreviations: bs, brephic shell; ha, halo; lse, larval setae; mas, mature shell; ms, metamorphic shell; mse, marginal mantle setae; np, nick points; ns, neanic shell; pr, protegulum; ps, post-metamorphic shell; te, tentacle

The patterns of early ontogeny here described for *Eohadrotreta* are almost identical to those of the early to middle Cambrian acrotretoid genera ‘*Homotreta*’, *Prototreta* and *Linnarssonia* recently described by [48]. The characteristic morphology of dorsal metamorphic shell with a posteromedially located protegulum and two pairs of dorsal larval setal sac impressions can be seen also on photographs of such genera as *Acrotreta*, *Dactylotreta*, *Longipegma* [59], *Neotreta*, *Picnotreta* [60] and *Hadrotreta* [61], etc. It suggests that this type of ontogeny was already established in the earliest Cambrian acrotretoids, including Chinese species of *Eohadrotreta*, and may have been retained within the group through the Cambrian Period (Fig. 7a**–**c).

The morphology of metamorphic shell of *Eohadrotreta* is also more or less identical to those described from paterinates like *Dictyonina*, *Micromitra* (Fig. 7g–h), *Olenekotreta* and *Paterina* ([26, 48]). Both acrotretoids and paterinates have a metamorphic shell that is ventribiconvex with a broad gap between the two valves along the posterior margin. The rudiments of the bivalved protegula are preserved as inflated dorsal and ventral posteromedial mounds. The dorsal valves exhibit two pairs of symmetrically arranged, inflated lobes, which according to [26] belong to the setal sacs containing sets of larval setae, homologous to those described by [20] for *Novocrania*. The paired ventral anterolateral lobes in paterinates were explained by ([26], text-Figure 3B) as impressions indicating possible position of the ventral digestive diverticulum, while the median cleft between them represents the trace of the junction of the ventral mesentery with the valve floor. It is likely that this explanation is also applicable to the pair of ventral lobes and median cleft documented for the acrotretide metamorphic shell.

Among the early Cambrian lingulides, the early ontogeny of *Eoobolus* described by [29, 48] is particularly interesting as its metamorphic shell also shows basic similarity to that of *Eohadrotreta* (Fig. 7f). Balthasar suggested that *Eoobolus* lacks a protegulum [29]. However, here we follow Ushatinskaya [48], who indicates the presence of the protegulum and two pairs of larval setal sac impressions on the dorsal metamorphic shell of *Eoobolus siniensis*. The ventral metamorphic shell of *Eoobolus* lacks lobations, but it possesses two symmetrical bundles of folds radiating from the posterior valve margin, which strongly resemble similar fine folds observed on the ventral protegulum of *Eohadrotreta* (Fig. 2a–g). The metamorphic shell of *Eoobolus* is covered by hemispherical pits, which also most probably are identical to those of *Eohadrotreta*. Thus, it is clear that at least some of the earliest lingulides possessed the slightly modified ‘paterinate’ type of metamorphic shell (Fig. 7d–f), previously described from paterinid *Micromitra* [26].

**Fig. 8:**
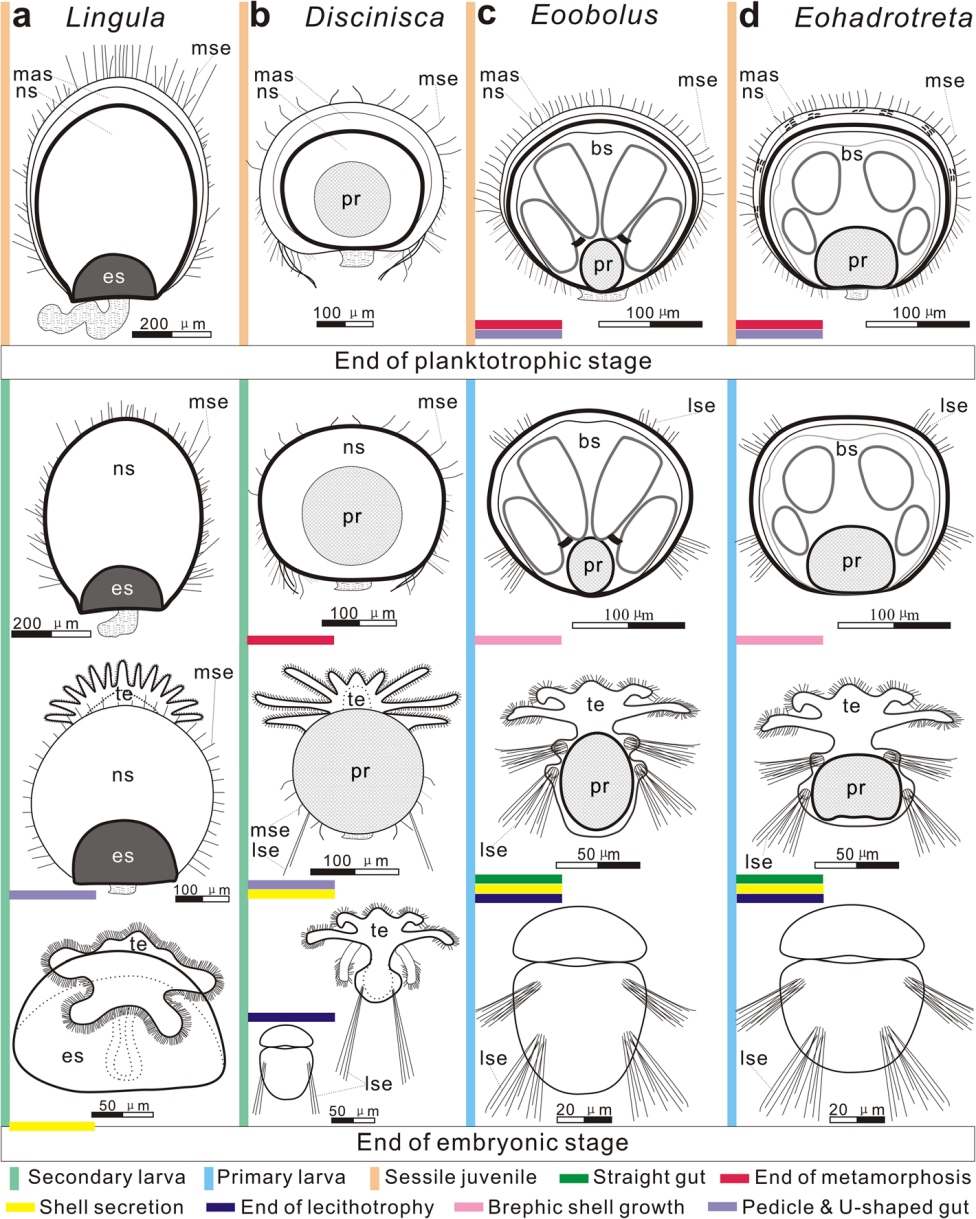
Life cycles, including earliest ontogenetic development stages, in different lingulate brachiopods. **a** extant *lingula*, showing life cycle of secondary larva. **b** extant *Discinisca*, showing life cycle of secondary larva. **c** early Cambrian *Eoobolus*, showing life cycle of primary larva with the completion of metamorphosis after settlement. **d** early Cambrian *Eohadrotreta*, showing life cycle of primary larva with the completion of metamorphosis after settlement (modified from [12, 15, 20, 26, 29, 45]). Abbreviations: bs, brephic shell; lse, larval setae; mas, mature shell; mse, marginal mantle setae; ns, neanic shell; pr, protegulum; te, tentacle

Remarkably, the ‘paterinate’ type larva also occurs in some Early Palaeozoic rhynchonelliform brachiopods, particularly in the orthoid *Notorthis* [33, 34] and gonambonitoid *Oslogonites* [36]. In both taxa the protegulum was secreted as the bivalved shell, while the dorsal brephic shell shows two pairs of setal sac impressions and a broad posterior gap between valves (Fig. 7l). Although, complex microtopography of the dorsal larval shell in these rhynchonelliforms suggest its flexibility and probably organic composition, the secretion of the mineralised shell fabric commenced on completion of metamorphosis after settlement. Remarkably, the size of the *Oslogonites* metamorphic shell (about 225 μm long and 290 μm wide) is within the range observed in the acrotretoids. Furthermore, two pairs of setal sacs on the dorsal metamorphic shell of *Eohadrotreta* are also comparable with that of Cambrian siphonotretid *Siphonobolus* [28] (Fig. 7d), orthotetid *Coolinia* [38] and even the enigmatic brachiopods *Mickwitzia* [29, 62] (Fig. 7i), *Salanygolina* [46] (Fig. 7j–k), although there are some variations mostly in the ventral metamorphic shells. The occurrence of identical larval types in distantly related brachiopod lineages like paterinates, acrotretoids, lingulids, siphonotretoids and different rhynchonelliforms suggests that the ontogenetic pattern, characterised by indirect development with a prolonged planktotrophic larval stage and the larval body plan are plesiomorphic for all major brachiopod lineages, and probably evolved within the stem group brachiopods (Fig. 7). It is also an indication that the major changes in the body plan, in particular the origin of an U-shaped digestive tract, which are completed in the recent representatives of the class Lingulata (lingulids and discinids) at the beginning of the pelagic stage (Fig. 8a, b). However, such significant changes were delayed in the earliest members of the clade until settlement, when metamorphosis had occurred (Fig. 8c, d).

Comparison with the earliest ontogeny of the recent discinids [15, 45], reveals several important differences. In *Discinisca* a single pair of larval (embryonic) setal sacs are already present in the embryo. They are shed shortly after the bivalved shell is developed [15] and replaced by sets of ‘curved’ and ‘flexible’ setae, attached to the mantle margin, which are morphologically adult setae [45]. Early after hatching, at the growth stage of 4 pairs of cirri, the *Discinisca* larva exhibits all major characters of the adult animal body plan and possesses a bivalved shell secreted by the mantle, functional intestine with an open anus, nephridia, adult curved setae and a pedicle, so it represents a typical secondary larva (Fig. 8b), unlike larvae of the acrotretoids *Eohadrotreta* and the lingulid *Eoobolus* (Fig. 8c, d). The discinid larva also possesses statocysts, which are probably apomorphic feature evolved later within the group in a process of adaptation to the prolonged pelagic part of life cycle. As was demonstrated by [30], the ontogenetic pattern observed in recent discinids is also present in the Early Ordovician representatives of the lingulide Family Paterulidae, the sister group of the Discinidae according to the phylogenetic analysis by [8]. Therefore, the secondary larva of discinids probably evolved already by the end of Cambrian. In the earliest Cambrian lingulates *Eohadrotreta* and *Eoobolus,* the adult body plan appeared upon completion of the pelagic part of the life cycle, as a result of metamorphosis, which occurred after settlement (Fig. 8c, d). However, the extant lingulids are characterised by direct development [45]. The freshly hatched *Lingula* shows all main features of the adult animal, including the shell, which is formed at the end of the embryonic stage [12]. Therefore, the lingulate life cycle underwent significant transformation through evolutionary history of the group, and the early pelagic stages of the extant discinids and lingulids are not directly comparable with the pelagic larvae of the early Cambrian linguliforms due to the significant heterochronic (peramorphic) modification of the life cycle (Fig. 8). Thus the shells of free-swimming juveniles during pelagic stage of the extant discinids and lingulids can be compared only to the neanic shells of almost all Early Palaeozoic brachiopods developed on completion of metamorphosis after settlement notwithstanding the planktotrophic or lecithotrophic nature of their larvae. Among extant brachiopods, the larva of the craniids retains some similarity with the acrotretid larva. In particular, the fully grown larva of *Novocrania* ([20], Figure 15) clearly shows the rudiment of dorsal protegulum flanked by two pairs of larval setal sacs (see also [29], p. 416). The illustrated pattern, probably, has some resemblance to the early acrotretoid larva at the stage of the initial shell secretion. However, since the larva of *Novocrania* is characterised by a lecithotrophic larval habit, with only the dorsal valve secreted by the larva, secretion of the ventral valve is delayed until the end of metamorphosis [31, 63].

## Conclusions

1. The early Cambrian acrotretoid *Eohadrotreta* was characterised by a planktotrophic larva and indirect development with a prolonged pelagic part of the life cycle.
2. The larval body plan of *Eohadrotreta* with a bivalved shell, two pairs of dorsal setal sacs and anterior–posterior alignment of the gut supported by long ventral mesentery was shared with lingulids, paterinates and some distantly related rhynchonelliforms, thus it was most probably plesiomorphic for the Brachiopoda.
3. Major axial rearrangements in the larval body plan resulted in the origin of the U-shaped digestive tract supported by lateral mesenteries and the anterolaterally placed anus, which occur in extant lingulides at the embryonic or early pelagic stage, were substantially delayed in the acrotretids and early Cambrian lingulides, like *Eoobolus*, hence they took place only on settlement during metamorphosis.
4. In comparison to the Early Palaeozoic linguliforms, ontogeny of the extant lingulids and discinids are strongly affected by heterochronic modifications, therefore free-swimming juveniles of the extant discinids and lingulids can be compared only with the acrotretoid neanic shell; also the embryonic shell of *Lingula* should be considered as a relatively recent novelty and it was not at all characteristic for the Palaeozoic brachiopods.

## Abbreviations

al: Anterior lobe
all: Anterolateral lobe
bs: Brephic shell
c: Shallow cleft
f: Pedicle foramen
ff: Fine fold
ha: Halo
ll: Lateral lobe
lse: Larval setae
mas: Mature shell
ms: Metamorphic shell
mse: Marginal mantle setae
np: Nick points
ns: Neanic shell
pmm: Posteromedian mound
pr: Protegulum
ps: Post-metamorphic shell
sp: Slope
su: Median sulcus
te: Tentacle
